# Adsorption Study for the Removal of Nitrate from Water Using Local Clay

**DOI:** 10.1155/2019/9529618

**Published:** 2019-02-03

**Authors:** A. Battas, A. El Gaidoumi, A. Ksakas, A. Kherbeche

**Affiliations:** Laboratory of Catalysis, Materials and Environment, Higher School of Technology, Sidi Mohamed Ben Abdellah University, 30000 Fez, Morocco

## Abstract

Our research aimed at the removal of nitrate ions through adsorption by local clay. A series of batch experiments were conducted to examine the effects of contact time, adsorbent characteristics, initial concentration of nitrate, pH of the solution, concentration, and granulometry of adsorbent. Adsorption isotherms studies indicated that local clay satisfies Freundlich's model. The rate of reaction follows pseudo-second-order kinetics. Local clay successfully adsorbs nitrates at pH acid. The adsorption capacity under optimal conditions was found to be 5.1 mg/g. The adsorption yield increases with adsorbent dose and decrease with initial concentration of nitrate. The local clay was characterized by the X-ray fluorescence method (XRF), X-ray diffraction (XRD), Fourier transform-infrared spectroscopy (FTIR), scanning electronics microscopy (SEM), and measurement of specific surface area (BET). The results of the study indicated that local clay is useful materials for the removal of nitrates from aqueous solutions which can be used in water treatment without any chemical modification.

## 1. Introduction

Water resources are heavily polluted by several nitrogen compounds, such as nitrates, nitrites and ammonium. Agricultural, industrial and household wastewaters are the major sources of nitrates in the surface and ground waters [1]. Many investigations related the high nitrates concentration in water to the eutrophication in aquatic environment [2]. Excess nitrates in drinking water could potentially cause human health problems such as blue-baby syndrome in infants and stomach cancer in adults [3]. World Health Organization (WHO) recommended concentration limit in the drinking water as to not exceed 50 mg/L [4].Therefore, numerous techniques for the removal of nitrates from water including adsorption, ion exchange, biological denitrification, chemical denitrification, electrodialysis and reverse osmosis, etc... [5]. Meanwhile, the adsorption is considered the most simple and efficient method [6, 7].

The application of local adsorbent for adsorption or elimination of nitrates present in wastewater is the object of the present study because of numerous economic advantages [8, 9]. In fact, clays are abundant and they present excellent physicochemical stability and structural and surface properties [10]. Our investigations were focused on the following aspects: (1) adsorption behavior of nitrate using local clay, including isotherms studies and kinetics, in batch experiments; (2) the effect of contact time, adsorbent characteristics, initial concentration of nitrate, pH of solution, dose and granulometry of adsorbent on adsorption process.

## 2. Materials and Methods

### 2.1. Materials

The adsorption tests were carried out with greenish clay (A_1_) rich in free silica (Quartz), found in large quantities in the northern region of Morocco. A synthetic solution of nitrate was prepared by dissolving 722 mg of potassium nitrate (KNO_3_) in 1 L of distilled water. KNO_3_, chlorhydric acid (HCl), and Sodium hydroxide (NaOH) were purchased from Sigma-Aldrich.

### 2.2. Adsorbent Characterizations

#### 2.2.1. X-Ray Fluorescence

The adsorbent chemical compositions were determined by the X-ray fluorescence, according to the method given by Ôzcan et al. [11]. The energy spectrum allows the determination of the amount of elements based on their characteristic peaks. Thus, 1g of adsorbent was preliminary heated to 110°C for 24 hours to remove water contained in the sample and is then calcined at 1000°C for 5 min to transform it in its oxides, which will be evaluated by percentage weight. Analysis of the chemical compositions was achieved by Oxford MDX 1000.

#### 2.2.2. X-Ray Diffraction (XRD)

XRD analysis is based on constructive interference of monochromatic X-rays and a crystalline sample. Powder diffractograms were measured on an X'Pert PRO Panalytical diffractometer (*λ*_Cu−K*α*_= 1.54060Å) equipped with an X'Celerator scintillation detector operating at 30 mA and 40 kV from 10° to 80° with a scanning rate of 2°.min^−1^.

#### 2.2.3. Fourier Transform-Infrared Spectroscopy (FTIR)

The vibrational behaviour of the respective samples was examined by Fourier transform infrared spectroscopy, in the range 400-4000 cm^−1^ with resolution of 4 cm^−1^, using FTIR spectrometer (Bruker Vertex 70). The samples were analysed in powder form (ATR technique).

#### 2.2.4. Scanning Electronic Microscopy (SEM)

The Scanning Electronic Microscopy is used to observe the texture of the clay sample and to characterize mineralogical assemblages. This analysis was carried out by Scanning Electronic Microscope, equipped with a probe EDAX, model QUANTA 200. The samples were analysed in pellet form.

#### 2.2.5. Measurement of the Specific Surface Area

In this work the measurement of the specific surface of the adsorbent A_1_ is very important to know the sites available for nitrate adsorption. The specific surface area was measured by the Brunauer-Emmett-Teller (BET) using AUTOSORB-1 system.

#### 2.2.6. Laser Granulometric Analysis

The laser granulometric analysis (Beckman Coulter LS) of adsorbent A_1_ has been determined by Fraunhofer method.

### 2.3. Dosage of Nitrate

In this work, we used the spectrometric method (UV-visible photolab 6600 spectrophotometer). In the presence of sodium salicylate, the nitrates give sodium paranitrosalicylate, colored yellow and able to be determinate by spectrophotometer.

### 2.4. Adsorption Capacity

The choice of an adsorbent is based on its adsorption capacity expressed generally in mg pollutant/g adsorbent, itself related to the size of the specific surface and the total pore volume [12, 13]. To study the adsorption capacity of nitrate ions on the adsorbent A_1_, 0.4 g of the adsorbent is introduced in 20 mL of a solution of nitrates (concentration of 100 mg/L), which corresponds to an initial charge of adsorbent of 20 g/L. Then, the mixture was stirred for 2 h on a magnetic stirrer (type SI Analytics GmbH) with the speed of 200 rpm at a temperature of 25°C before to be filtered through at 0.45 *μ*m filter membrane for analysis. The residual concentration of each sample allows determining the quantity of fixed nitrate ions per gram of adsorbent according to

(1) $$\begin{array}{c} q_{t}=\frac{\left(C_{0}-C_{t}\right)*V}{m} \end{array}$$

where, q_t_ is the amount of adsorbed ions per unit adsorbent at instant t (mg/g), m is the mass of adsorbent used (g), C_0_ represents the initial concentration of nitrate ion in solution (mg/L), C_t_ is the concentration of nitrate ion in solution at instant t (mg/L) and V is the volume of solution used (L).

It is also possible to evaluate the quantity adsorbed by the adsorption yield given by

(2) $$\begin{array}{c} R\;\;\left(\;\%\;\right)=\frac{\left(C_{0}-C_{t}\right)}{C_{0}} \end{array}$$

where C_0_ and C_t_ are the concentrations of nitrate at initial condition and at any time, respectively.

### 2.5. Adsorption Kinetics

The adsorption kinetics reflects the evolution of the adsorption process as a function of time, this is a key parameter considered when selecting an adsorbent [14]. Rapid adsorption is recommended for treatment methods using adsorption as a purification process [15]. Several models describing the diffusion of solutes at the surface and in the pores of the particles have been developed (film distribution model, intraparticulate diffusion model, diffusion model extra-particulate, pore diffusion model, etc.) [16]. However, in the case of nitrates, simplified models such as pseudo-first-order kinetics and pseudo-second-order kinetics were popular.

#### 2.5.1. Pseudo First-Order Model

Lagergren showed that the adsorption rate of aqueous solution on the adsorbent is based on the adsorption capacity and following a first-order equation [17]. The nonlinear form of the first-order equation is given by

(3) $$\begin{array}{c} \frac{dq_{t}}{dt}=k_{1}\left(\;q_{e}-q_{t}\;\right) \end{array}$$

where q_t_ (mg.g^−1^) is the adsorption capacity at time t, q_e_ (mg.g^−1^) is the equilibrium adsorption capacity, and k_1_ (min^−1^) is the first-order constant.

After integration and application of the initial condition of q = 0 at t = 0, the equation takes the following form:

(4) $$\begin{array}{c} \log\left(\;q_{e}-q_{t}\;\right)=\log q_{e}-\frac{k_{1}t}{2\text{,}303} \end{array}$$

#### 2.5.2. Pseudo Second-Order Model

The expression of the second-order was used to describe the kinetics of the adsorption processes. The pseudo second-order kinetic model equation is expressed as follows:

(5) $$\begin{array}{c} \frac{dq_{t}}{dt}=k_{2}{\left(\;q_{e}-q_{t}\;\right)}^{2} \end{array}$$

where k_2_ (g.mg^−1^ min^−1^) is the rate constant of the pseudo second order.

The integration of this equation to t = 0 and q_t_ = 0 takes the linear form

(6) $$\begin{array}{c} \frac{t}{q_{t}}=\frac{1}{k_{2}{q_{e}}^{2}}+\frac{t}{q_{e}} \end{array}$$

### 2.6. Adsorption Isotherms

The adsorption isotherms are used to study the adsorption mechanisms. An adsorption isotherm describes the equilibrium at a given temperature between the adsorbed solute concentration and the unadsorbed concentration [18]. However, the literature shows that authors pay less attention to the type of isotherm obtained; they focus more on the mathematical model that translates the experimental results. Thus, two models are widely used: the Langmuir model (7) and the Freundlich model (8):

(7) qe=qmb.Ce1+b.Ce

(8) qe=Kf.Ce1/n

where q_e_ (mg/g) is an adsorbed amount at equilibrium; C_e_ (mg/L) is a concentration at equilibrium; q_m_ (mg/g) is a maximum adsorption capacity of the monolayer; K_f_ and n are the parameters of Freundlich; b is the parameter of Langmuir.

The Langmuir model assumes that (1) the adsorption is monolayer, (2) it is reversible, (3) each site can adsorb only one ion, and (4) there is no interaction between the ions with adsorbent. The Freundlich model suggests that binding energy decreases exponentially with increasing surface saturation. This model is widely used, although it is empirical. It gives an indication of surface heterogeneity [19].

#### 2.6.1. Freundlich Isotherm

The linear form of the equation of Freundlich can be written in logarithmic form according to the following relation:

(9) $$\begin{array}{c} \log q_{e}=\log K_{f}+\frac{1}{n}\log C_{e} \end{array}$$

where K_F_ and 1/n are the constants of Freundlich connected to the adsorption capacity and the intensity of adsorption of the adsorbent, respectively.

#### 2.6.2. Langmuir Isotherm

By applying the Langmuir hypothesis, it can be concluded that in the case of a large quantity of adsorbed solute, the parameter b, C_e_ becomes considerably greater than 1. This implies that q tends to q_m_ [20]. The linear form of the Langmuir equation is

(10) $$\begin{array}{c} \frac{C_{e}}{q}=\frac{1}{q_{m}.b}+\frac{C_{e}}{q_{m}} \end{array}$$

### 2.7. Study of the Parameters Effect

#### 2.7.1. Adsorption Tests

The adsorption tests were carried out in batch mode in beakers containing 20 mL of the solution to be treated, mounted on a thermoregulated agitating platform. A solution of KNO_3_ was used as a source of nitrate ions under the following working conditions: the initial concentration of NO_3_^−^ varies from 50 to 200 mg/L, the adsorbent dose varies from 5 to 40 g/L, and the pH is from 2 to 9, with a contact time set at 120 min and a stirring speed of 200 rpm at constant temperature of 25°C. Finally, the solutions were filtered and the filtrate was recovered for analysis.

#### 2.7.2. The Adsorbent Dose Effect

The effect of the adsorbent filler on the retention of nitrate ions was studied for the values of 5, 10, 20, and 40 g/L of adsorbent A_1_while keeping the other parameters fixed (room temperature, agitation speed of 200 rpm, and the initial nitrate concentration is 100 mg/L).

#### 2.7.3. The Initial Concentration Effect

In order to study the effect of the initial concentration on the adsorption of nitrate ions, variable NO_3_^−^ concentrations (50, 100, 150, and 200 mg/L) with an adsorbent load of 20 g/L of adsorbent A_1_ were used.

#### 2.7.4. The pH Effect

The pH is an important parameter to be considered in sorption processes because it may affect both the properties of the adsorbent and the composition of the solution [21].The study of the pH effect on the adsorption of nitrate ions was carried out by varying the pH from 2 to 9 with an initial concentration of NO_3_^−^ ions (100 mg/L) and adsorbent dose fixed at 20 g/L at room temperature. The initial pH of the solutions was measured by a pH-meter and adjusted to the desired values using 0.01N HCl and NaOH solutions.

#### 2.7.5. Adsorbent Granulometry Effect

The effect of the granulometry was studied by the use of adsorbent with particle sizes: d<110*μ*m, 200 *μ*m<d <400 *μ*m, and d > 400 *μ*m.

## 3. Results and Discussion

### 3.1. Characterization of Adsorbent

#### 3.1.1. Chemical Compositions of Adsorbent by X-Ray Fluorescence

To determine the chemical compositions of studied adsorbent, we conducted a spectrometry analyses XRF.  Table 1 shows the material analyses results by X-ay fluorescence spectrometry.

The analyses carried out on the investigated adsorbent (Table 1) show that the mass ratio SiO_2_/Al_2_O_3_ is 4.24. This high value suggests the presence of a large amount of free silica (Quartz). A_1_ showed 7. 80 % of loss-on-ignition (LOI) may be due to the dehydroxylation of the clay and removal of its organic and carbonate compounds.

#### 3.1.2. X-Ray Diffraction Analysis

**Figure 1:**
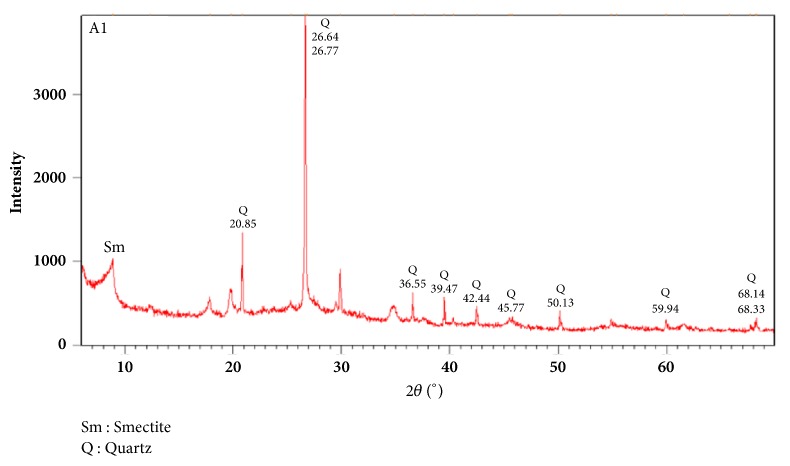
XRD spectrum of adsorbent A_1_.

A mineralogical analysis of the raw sample of adsorbent A_1_ was achieved by X-ray diffraction in order to identify the main adsorbent minerals. The diffractogram of the adsorbent sample A_1_ (Figure 1) showed that this material is mostly composed of quartz with peaks located at 2*θ*-angles (°): 20.85, 26.77, 36.55, 39.47, 42.44, 45.77, 50.13, 59.94, and 68.33.

#### 3.1.3. Fourier Transforms Infrared Spectroscopy Analysis

**Figure 2:**
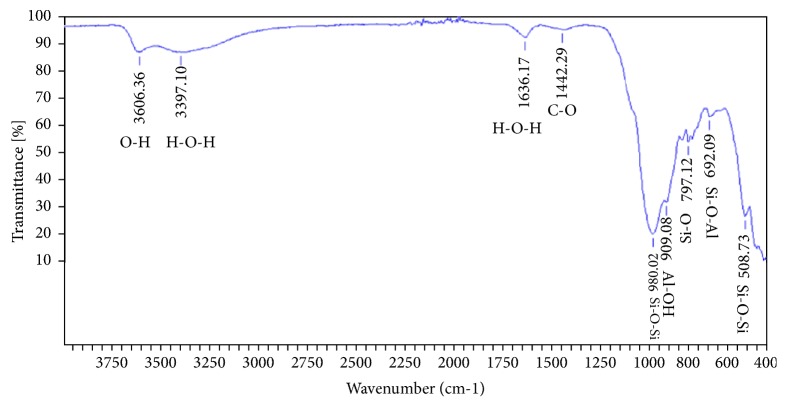
FTIR spectrum to Fourier transforms of adsorbent A_1_.

The FTIR spectroscopy analysis gets absorption bands corresponding to the various vibrations of the characteristic bonds of the phases already detected by XRD. The FTIR spectrum of adsorbent A_1_ (Figure 2) showed several absorption bands the peak to 3606 cm^−1^ assigned to the vibration band of the hydroxyl group. Broad bands located at 3397 and 1636 cm^−1^ are the cause of the axial and angular deformation of water molecules adsorbed between sheets [22]. The centred strips at 797 and 1442 cm^−1^ correspond to the stretching of the vibrations of Si-O in SiO_2_ and CO_3_, respectively, while the band at 508 cm^−1^ is probably assigned to the vibrations of the connections Si-O-Si of quartz. Vibration bands located at 692 and 909 cm^−1^are assigned to deformation of Si-O-Al and Al-OH, respectively.  Table 2 represents the vibration bands and their assignments for adsorbent A_1_.

#### 3.1.4. Measurement of the Specific Surface Area

The specific surface area and total pore volume of the adsorbent A_1_were determined as 38.08 m^2^ g^−1^ and 8.75 cm^3^ g^−1^, respectively, a bit larger than that of purified montmorillonite (34 m^2^.g^−1^) of commercial Argentine bentonite [23]. We displayed in Table 3 features of the present adsorbent compared to those of some other adsorbents reported in the literature.

#### 3.1.5. Laser Granulometric Analysis

**Figure 3:**
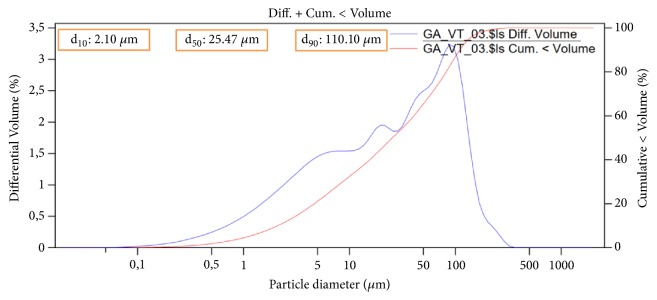
Granulometric distribution of adsorbent A_1_.

The particle size, shape and distribution have been reported to be a very important property in determining the industrial uses of clays [27]. For this purpose, Beckman Coulter LS Particle Size Analyzer was used to determine the granulometric parameters d_10_, d_50_, and d_90_ of the local clayA_1_. The cumulative and differential particle size distributions of this material are presented in Figure 3 resulting in 2.10, 25.47, and 110.10 *μ*m, for d_10_, d_50_, and d_90_, respectively. The effect of the adsorbent granulometry on the adsorption of nitrate will be presented in Section 3.4.4.

#### 3.1.6. Scanning Electron Microscopy (SEM)

**Figure 4:**
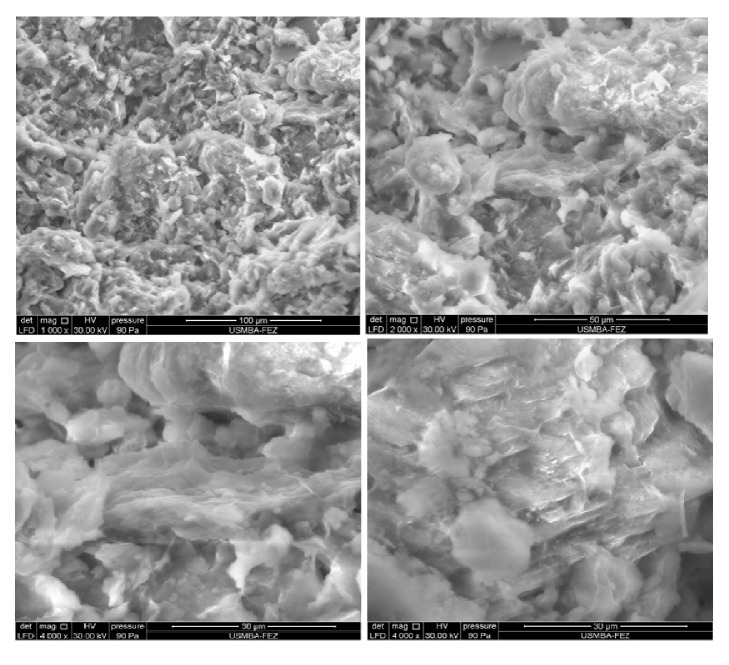
SEM images of adsorbent A_1_with three different resolutions 100, 50 and 30 *μ*m.

The images obtained by scanning electron microscopy of the adsorbent sample with three different magnifications are shown in Figure 4. The image of the local clayA_1_ indicates that the adsorbent particles are in the form of platelets in the pace of leaves with irregular contours [28, 29]. This is a morphology encountered both for Kaolinites poorly crystallized only for Illites as observed by Konan et al. [30].

### 3.2. Adsorption Kinetics

#### 3.2.1. Contact Time

**Figure 5:**
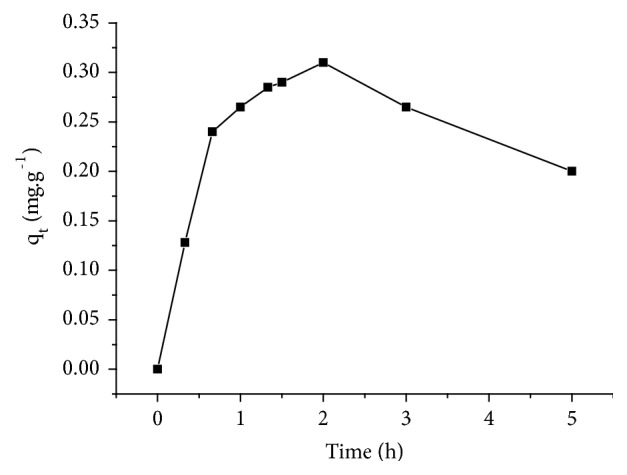
Evolution of the adsorption capacity of nitrates on adsorbent A_1_.

The capacity adsorption of nitrate ions solution 100 mg/L on an adsorbent dose 20 g/L was followed as a function of time (h).The results presented in Figure 5 show that the adsorption capacity of the nitrate ions increases with contact time and reaches a maximum value at 2 h. After 2 h, the adsorption capacity decreases. This decrease can be explained by the saturation of the free adsorbent sites and may be due to an adsorbate release. According to the Freundlich hypothesis, the binding energy decreases exponentially with increasing surface saturation [31].

#### 3.2.2. Pseudo First-Order Model

**Figure 6:**
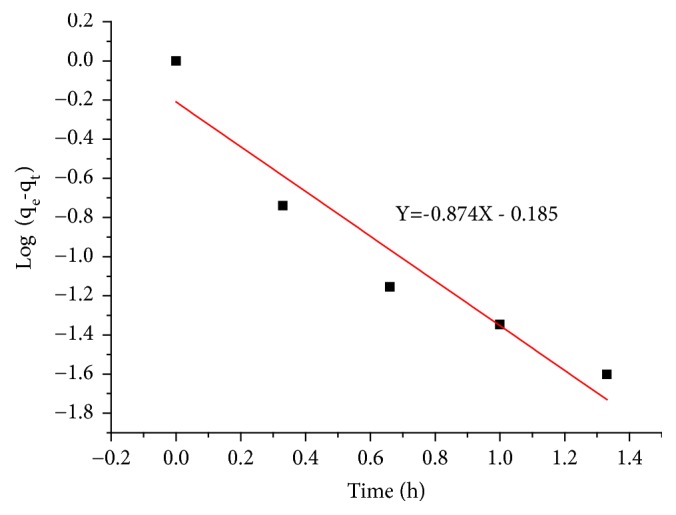
The adsorption kinetics of the nitrate ions on the adsorbent A_1_ according to the pseudo-first-order kinetic model.

The reaction order is determined by the following operating conditions: the nitrate concentration solution of 100 mg/L, the adsorption dose of 20 g/L, the temperature of 25°C, and the stirring speed of 200 rpm.  Figure 6 represents the adsorption kinetics of the nitrate ions on the adsorbent A_1_ according to the pseudo-first-order kinetic model. The linear equation is presented by (4).

#### 3.2.3. Pseudo Second-Order Model

**Figure 7:**
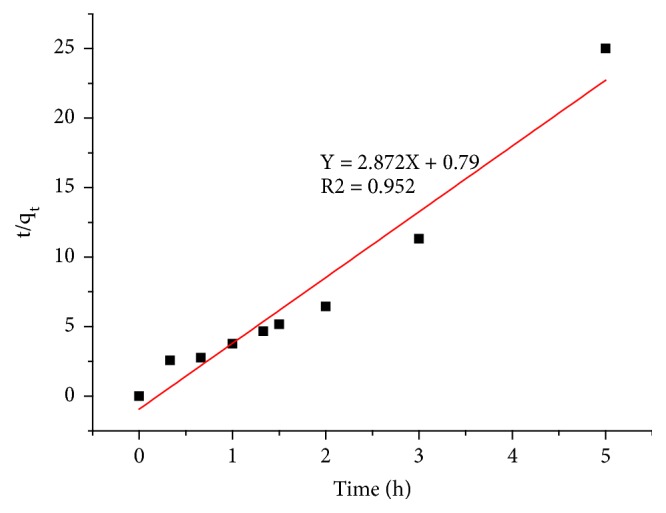
The adsorption kinetics of the nitrate ions on the adsorbent A_1_ according to the pseudo second-order kinetic model.

The Figure 7 shows the adsorption kinetics of the nitrate ions on the adsorbent A_1_ according to the pseudo-second-order kinetic model. The linear equation is presented by (6).  Table 4 indicates that the adsorption kinetics of nitrate ions onto the adsorbent A_1_is better described by the pseudo second-order model. The correlation coefficient is close to unity (R^2^ = 0.952), and the value of q_e_ calculated by the pseudo second-order model is very similar to that determined experimentally.

### 3.3. Adsorption Isotherms

**Figure 8:**
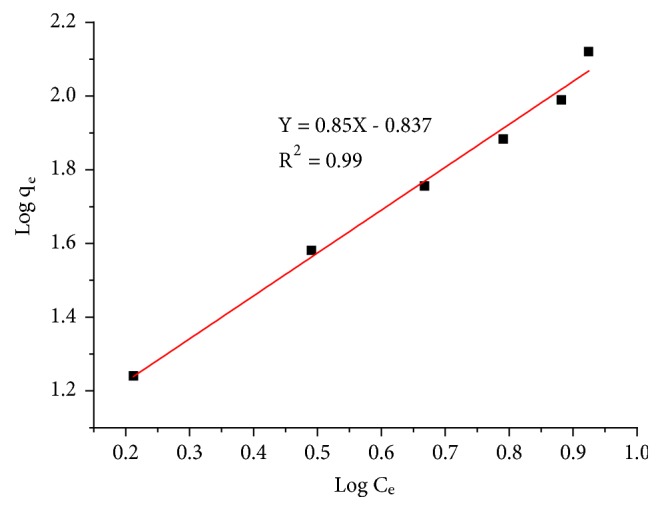
Adsorption Isotherm of the nitrate ions onto the adsorbent A_1_ according to the Freundlich model.

**Figure 9:**
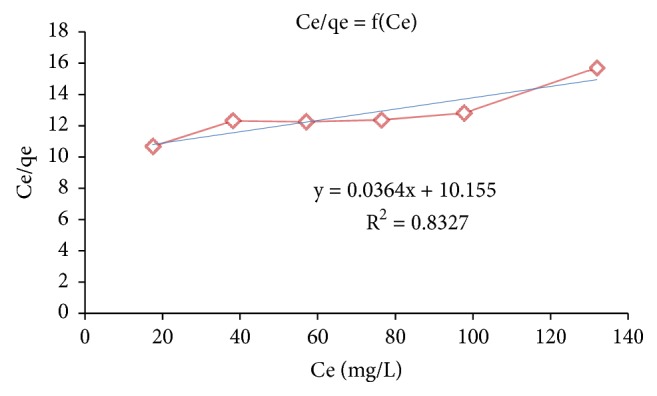
Adsorption isotherm of the nitrate ions onto the adsorbent A_1_ according to the Langmuir model.

The adsorption isotherms of nitrate ions onto the adsorbent A_1_ is studied by the representation of the equilibrium adsorption capacity variation q_e_ (mg/g) as a function of the equilibrium concentration of nitrate (mg/L), according to the Freundlich and Langmuir models, represented by Figures 8 and 9 respectively. The obtained results are grouped in Table 5.

From the results shown in Table 5, it can be said that the adsorption of nitrate ions on the adsorbent A_1_ is best described by the Freundlich model (correlation factor close to the unit R^2^ = 0.99). According to Freundlich hypotheses, the adsorption energy of nitrate ions on the adsorbent A_1_ decreases exponentially with increasing surface saturation and the surface of the adsorbent is heterogeneous [32, 33].

### 3.4. Parameters Effects on Adsorption of Nitrate Ions

#### 3.4.1. Effect of Adsorbent Dose

**Figure 10:**
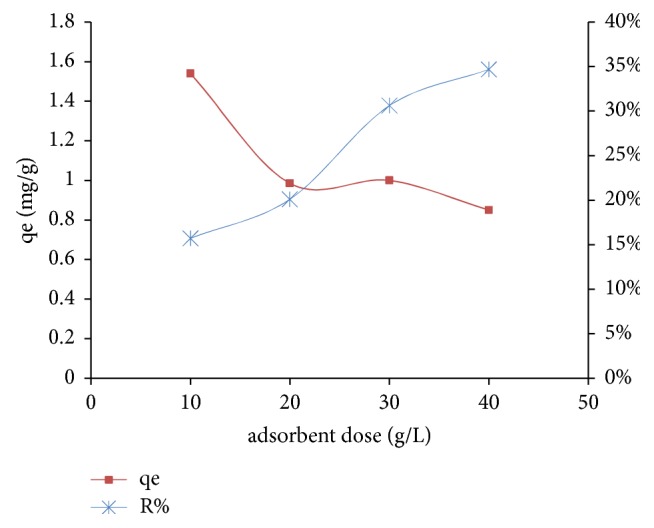
Effect of the adsorbent dose (adsorbent A_1_) on the adsorption of nitrate ions.

The adsorbent dose effect was investigated at room temperature for an initial concentration of NO_3_^−^ (100 mg/L) with stirring at 200 rpm for 120 min. The variation influence of the adsorbent dose is shown in Figure 10.

Figure 10 shows that the adsorption efficiency increases with increasing the adsorbent dose. This can be explained by the increase in the number of active adsorption sites, and the availability of the adsorption sites increases the adsorption capacity. The adsorption yields vary from 16 to 35% and better adsorption efficiency of 35% is observed for a concentration of adsorbent A_1_ at 40 g/L.

Figure 10 also shows the adsorption capacity (mg/g) of NO_3_^−^ depending on the adsorbent support in the solution. The curve follows a downward trend, indicating that the amounts of NO_3_^−^ adsorbed by the adsorbent A_1_ are inversely proportional to the adsorbent dose. Indeed, it is noted that q_e_ reaches a maximum of 1.54 mg/g for an adsorbent concentration equal to 10 g/L, while the lowest q_e_ is 0.7 mg/g, for an adsorbent concentration of 40 g/L.

#### 3.4.2. Initial Nitrate Concentration Effect

**Figure 11:**
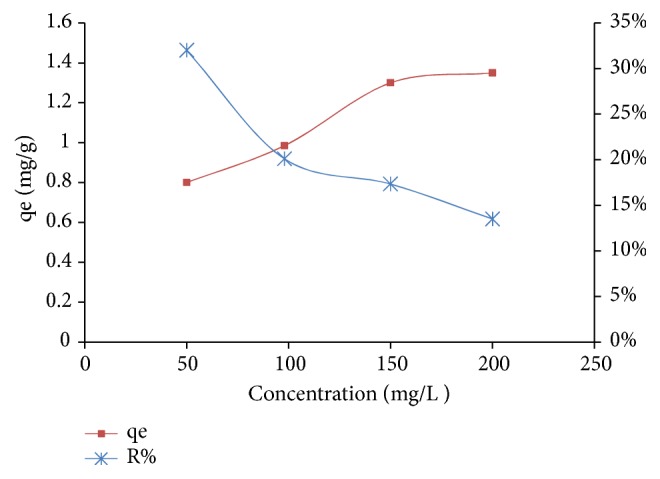
Effect of the initial concentration of NO_3_^−^.

The initial nitrate concentration effect was investigated at NO_3_^−^ concentrations (50, 100, 150, and 200 mg/L), and the adsorption tests were carried out at room temperature with stirring at 200 rpm for 120 min with 20 g/L of adsorbent A_1_.  Figure 11 shows the results of the yield and adsorption capacity as a function of the initial concentration of the nitrate ions.

The adsorption efficiency evolution as a function of the initial concentration of NO_3_^−^ follows a decreasing curve and thus the highest yields are observed for the lowest initial NO_3_^−^ concentrations. Indeed, the highest adsorption yield of nitrate ions (32%) is observed for an initial concentration of NO_3_^−^ in the solution equal to 50 mg/L. This yield decreases from 32% to 14% by varying the initial concentration of nitrate ions from 50 to 200 mg/L. That is, the adsorption capacity increases from 0.8 mg/g to 1.35 mg/g.

#### 3.4.3. pH Effect

**Figure 12:**
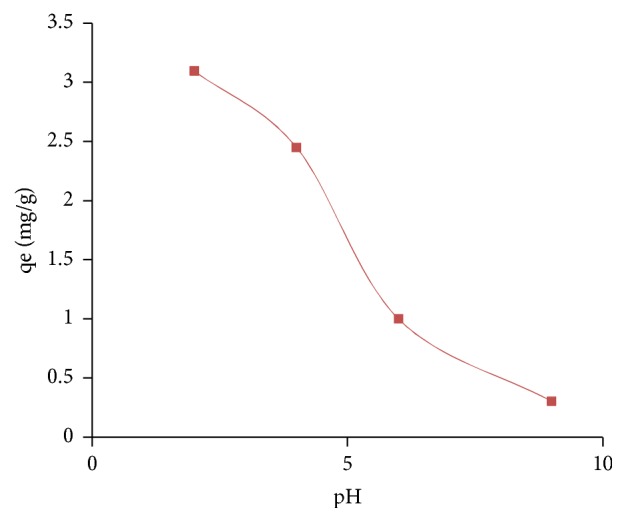
pH effect on the adsorption of NO_3_^−^.

The pH effect on the adsorption of nitrate ions was investigated by carrying out adsorption tests over a pH range from 2 to 9. The results are shown in Figure 12.

The pH of the solution is a very important parameter in the study of the adsorption of nitrate ions by clay. Figure 12 shows the variation of the adsorption capacity of NO_3_^−^ depending on the pH of a solution containing 100 mg/L of NO_3_^−^ and 20 g/L of the A_1_adsorbent. It is observed that the adsorption capacity q_e_ reaches their maximum at pH 2 and decrease with the increase in pH. Usually, the influence of pH on anion exchange reaction was mainly due to the competition between the hydroxyl ions and anions. When pH of the solution increases, the surface becomes negatively charged and the adsorption capacity for nitrate decreases, because negatively charged surface sites on the adsorbent did not favor nitrate due to the electrostatic repulsion [34, 35].

#### 3.4.4. Adsorbent Granulometry Effect

**Figure 13:**
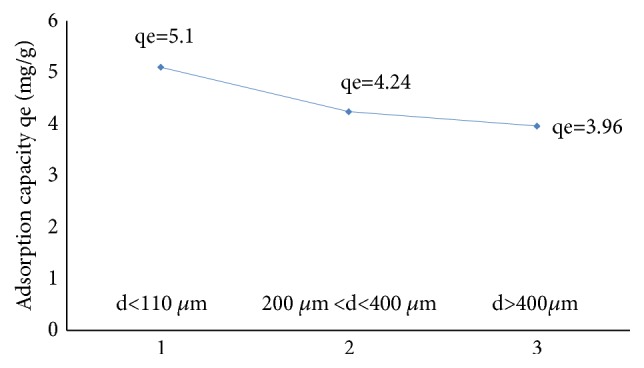
Effect of particle size on the adsorption of NO_3_^−^ions onto A_1_.

The effect of the particle size distribution on the adsorption of nitrate is represented by Figure 13. The results show that the particle size of the adsorbent A_1_ influences the adsorption capacity of the nitrate ions. The adsorption capacity reaches a maximum value for a granulometry of the adsorbent of d <110 *μ*m. This can be explained by the increase of the specific adsorption sites [36].

## 4. Conclusions

This study investigated the adsorption characteristics of local clayA_1_ as a potential adsorbent for the removal of nitrate from aqueous solutions using batch system. The experimental parameters were very important in order to understand the mechanism of adsorption of nitrate ions, such as the initial nitrate ion concentration, adsorbent dose, pH, and particle size. The results showed that the adsorption efficiency increased with the adsorbent dose and decreases with the initial concentration. The pH optimum for nitrate ions adsorption onto the adsorbent A_1_ is between 2 and 4. The adsorption capacity reaches a maximum value for the adsorbent granulometry from d <110*μ*m. Adsorption equilibrium applied by Langmuir and Freundlich allowed us to conclude that Freundlich's model shows the best correlation. The adsorption kinetics of nitrate ions on the adsorbent A_1_ is better described by the pseudo second-order model. The choice of this clay presents the opportunity to benefit from this cheap material found in nature with significant quantities.

## Figures and Tables

**Table 1:** Chemical compositions of the adsorbent A_1_in (%).

**Components**	**SiO**_**2**_	**Al**_**2**_ **O**_**3**_	**Fe**_**2**_ **O**_**3**_	**CaO**	**MgO**	**SO**_**3**_	**K**_**2**_ **O**_**3**_	**Na**_**2**_ **O**	**LOI**
**Wt. (**%**)**	63.00	14.85	5.02	1.11	2.65	0.06	5.14	0.06	7.80

**Table 2:** Infrared analysis results of different vibration bands.

**Adsorbent **	**Vibration bands (cm** ^**-1**^ **)**	**Assignment**
**Adsorbent A**_**1**_	3606	Vibration of the hydroxyl group
	3397-1636	Axial and angular deformation of water molecules
	1442	Vibration stretch of CO_3_
	909	Deformation of Al-OH
	797	Vibration stretch of Si-O
	692	Deformation of Si-O-Al
	508	Vibration of links Si-O-Si

**Table 3:** Comparison of BET surface area of local adsorbent studied A_1_ with other adsorbents.

**The sample**	**Specific surface in m** ^**2**^ **.g** ^**-1**^	**References **
**Montmorillonite**	34	[23]
**Smectite **	31.13	[24]
**Illite**	25	[25]
**Kaolinite**	21	[26]
**Adsorbent A**_**1**_	38.08	Present study

**Table 4:** Kinetic parameters of pseudo-first order and pseudo-second-order models.

**Adsorbent**	**Pseudo first order**			**Pseudo second order**		
	k_1_ (h^−1^)	qe (mg/g)	R^2^	k_2_ (g/mg.h)	qe (mg/g)	R^2^
**Adsorbent A**_**1**_	2.014	0.516	0.937	10.542	0.384	0.952

**Table 5:** Parameters of isotherm models for the nitrate ions adsorption onto the adsorbent A_1_.

**Adsorbent**	**Freundlich model**			**Langmuir model**		
	K_f_	n_f_	R^2^	b	q_m_ (mg/g)	R^2^
**A**_**1**_	0.145	1.176	0.990	3.54*∗*10^−3^	27.77	0.8327

## Data Availability

The data used to support the findings of this study are available from the corresponding author upon request.
